# Parasites of Native and Invasive Rodents in Chile: Ecological and Human Health Needs

**DOI:** 10.3389/fvets.2021.643742

**Published:** 2021-02-11

**Authors:** Carlos Landaeta-Aqueveque, Lucila Moreno Salas, AnaLía Henríquez, María C. Silva-de la Fuente, Daniel González-Acuña

**Affiliations:** ^1^Facultad de Ciencias Veterinarias, Universidad de Concepción, Chillán, Chile; ^2^Facultad de Ciencias Naturales y Oceanográficas, Universidad de Concepción, Concepción, Chile; ^3^Facultad de Medicina Veterinaria, Universidad San Sebastián, Concepción, Chile; ^4^Facultad de Ciencias Veterinarias, Universidad Austral de Chile, Valdivia, Chile

**Keywords:** rat, parasite, biological invasion, native (autochthonous), human health, spillover, vector-borne disease, zoonotic diseases

## Abstract

Invasive populations are a threat to biodiversity, resulting in the loss of species, and also a threat to human health, participating in the reservoir of diseases. Rodents are among the most important invasive species worldwide. Chile is a country that features island conditions in terms of geography and has been widely invaded by allochthonous rodents. In this mini-review, we updated the literature on macro-parasites infecting both native and invasive rodents and of vector-borne pathogens in continental Chile in order to assess the relative importance of invasive rodents from both ecological and public health points of view. A total of 174 parasite species were found, with *Siphonaptera* representing the most diverse group. When examining how parasites are shared between native and introduced rodents, the analysis suggests that parasites circulate freely within recipient populations, and are not significantly transmitted from source populations. Further, generalist parasites are typically more prone to being shared between native and introduced rodents. Most zoonotic parasites were reported in invasive rodents, suggesting that these rodents must represent a public health concern. Although several vector-borne pathogens have been reported in rodents or ectoparasites, most of the recently emerging research has illustrated that there is a lack of evidence on rodent–vector-borne zoonoses in most pathogens.

## Introduction

Invasive populations represent one of the most important threats to biodiversity (1), either interacting directly with native fauna (2), or by sharing their co-introduced parasites with them (3, 4). Invasive animal populations can also serve as a threat to human health, either co-introducing zoonotic parasites (5) or hosting previously existing zoonotic parasites (6, 7). Thus, increased knowledge related to parasitism and the processes driving parasite distribution among native and introduced animals is not only a biological conservation, but also, a public health issue. Rats (*Rattus* spp.) and mice (*Mus musculus*) are among the most spread invasive mammals around the world and constitute an important source of pathogens for humans (8, 9).

Chile is a country with island conditions given its geographic border, which includes the Los Andes Mountain Range, the Atacama Desert, and the Pacific Ocean, each of which clearly separates an invaded territory. Rats and mice were introduced into and have colonized the country, invading several environments and harboring zoonotic pathogens (10, 11); however, studies on zoonotic macroparasites have remained neglected over the last few decades, as studies related to vector-borne zoonoses (by microparasites) have been the main focus in public health. Conversely, studies focusing on the biodiversity and ecology of macroparasites among introduced rodents have increased slightly in recent years (12, 13). Thus, the aim of this study was to review and quantitatively explore the literature on macroparasites of rodents from the perspectives of public health and ecology. This review revisits the raw data from previously published articles to explore future pathways for the study of parasites in the inter-phase of native and introduced rodents.

## Methods for the Review and Analyses

A literature search of reports prior to December, 2020, was performed in PubMed (http://www.ncbi.nlm.nih.gov/pubmed/) and Scholar Google (https://scholar.google.com/) using the following terms: Acari, Ixodida, Phthiraptera, Siphonaptera, Nematoda, Cestoda, Trematoda, Acanthocephala plus Rodentia, and Chile. These taxa names were also replaced by their respective colloquial terms. In the case of ticks and fleas, the search considered literature published from 2005 and 2014, respectively. For previously published literature pertaining to those taxa, we referred to published reviews (14, 15). We also considered literature on the presence of these parasites in humans, as well as pathogens transmitted by these parasites. Given that the separation of the rodent species *Abrothrix longipilis* and *Abrothrix hirta* is rather new, we advise that some mentions of *A. longipilis* can correspond to *A. hirta*. When the parasite was identified only at the genus level, it was considered in this review when this was the sole report available on this genus, unless the evidence supports that they are different to other species of the same genus. In the case of the *Androlaelaps* sp. reported in *Abrothrix olivacea*, we considered it to belong to the *Androlaelaps farenholzi* complex given that it is morphologically within the morphospecies (16).

For ecological approximations, we assessed whether host specificity is a factor that affects the sharing of parasites between native and introduced rodents, as determined by two classifications. The first classification is dichotomous in nature; if a parasite is collected from myomorph and hystrichomorph rodents, it was considered a generalist (non-host-specific) parasite, while if it was only collected from one suborder of rodent, it was considered a specialist (host-specific) parasite. The second, more quantitative, classification was reflected by the number of hosts species from which the parasite was collected (i.e., the smaller the number, the more host-specific the parasite). We also explored whether the spillover propagule pressure (13) is a factor driving the structure of the parasitic supra-populations. To determine this, we assessed the associations between the mean abundance (MA) and prevalence of a parasite species in the source host populations (i.e., native hosts for native parasites, and introduced hosts for introduced parasites), and those (MA and prevalence) in the recipient host populations (native hosts for introduced parasites, and vice versa). If the spillover propagule pressure is significant, a positive correlation is predicted. We considered the raw data from one previously published study (17) in order to achieve this and we assessed the association using Spearman's correlation test.

## Parasites

One trematode (Plagiorchiida), eight cestode (Cyclophyllidea), 35 nematode (Trichinellida, Rhabditida, Strongylida, Ascaridida and Spirurida), 92 flea (Siphonaptera), 17 louse (Phthiraptera), 16 mite (Mesostigmata, Trombidiformes and Sarcoptiformes), and five tick (Ixodida) species were reported (12–70). Supplementary Table 1 **(helminths) and** **Supplementary Table 2** **(arthropods)** in Supplementary Material detail the species and hosts from which they have been collected.

## Zoonotic Parasites

Some of the reported helminth species frequently affect humans worldwide: *Hymenolepis* (*Rodentolepis*) *nana, Hymenolepis diminuta, Trichinella spiralis*, and *Calodium hepaticum* (71–74). There are reports of human infection with *Hymenolepis* spp. and *T. spiralis* in Chile (26, 75), but we did not find reports of human infection with *C. hepaticum* in this country. Other helminths, such as *Hydatigera taeniaeformis, Syphacia obvelata, Syphacia muris*, and *Trichuris muris*, have been rarely reported in humans (76–80) and they have not been reported in humans in Chile. As such, they are of lower public health concern. *Echinococcus granulosus*, which is an important zoonosis in Chile (81, 82), has been described only once in a rodent, indicating that rodents seem to be of minimal concern for this zoonosis.

*Rhipicephaus sanguineus, Ornithonyssus bacoti*, and *Pulex irritans* are the sole tick, mite, and flea species to parasitize rodents and humans; conversely, *Nosopsyllus fasciatus, Leptopsylla segnis*, and *Echidnophaga gallinacea* were reported to be aberrant parasites of humans (14, 15, 83).

## Vector-Borne Zoonoses

Few vector-borne pathogen have been reported to cause disease and have been detected in humans, vectors and in rodents in Chile. *Candidatus* Orientia chiloensis has been found in the chigger mite *Herpetacarus* eloisae parasitizing the rodents *A. olivacea, Abrothrix sanborni*, and *Geoxus valdivianus* (84, 85). There have been no reports related to the mites parasitizing humans. *Trypanosoma cruzi* has been found in native and invasive rodents and in the triatomine vectors *Triatoma infestans, Mepraia spinolai, Mepraia gajardoi*, and *Mepraia parapatrica* (86). To the best of our knowledge, there is no report of these triatomines parasitizing rodents in natural conditions in Chile; thus, analyses of the feeding profiles of the vectors are the main evidence supporting that they parasitize rodents (87). This disease is largely reported in humans in Chile (88).

The DNA of *Bartonella tribocorum* has been detected in *N. fasciatus* and *Plocopsylla* sp. *Bartonella mastomydis* has been detected in *Xenopsylla cheopis*, and *Bartonella* sp. has been detected in *X. cheopis, L. segnis, N. fasciatus, Hectopsylla* sp., *Neotyphloceras chilensis, Neotyphloceras pardinasi*, and *Sphinctopsylla ares* (12, 89). *Bartonella tribocorum* and *Bartonella rochalimae* have been detected in *Rattus rattus* and *Abrothrix* sp. in Chile; however, *B. rochalimae* has not been found in fleas isolated from rodents in Chile, but has been found in *P. irritans* from dogs (90). Only *B. tribocorum* and *B. rochalimae* have reportedly affected humans elsewhere, while rodents have been reported as the reservoir (91). Conversely, although zoonotic *Bartonella clarridgeiae* and *Bartonella henselae* have been detected in *Ctenocephalides felis* from cats (90), there is no evidence to suggest that rodents participate in their reservoir.

Regarding rickettsias, the DNA of *Candidatus* Rickettsia senegalensis has been detected in *Chilopsylla allophyla*, parasitizing *A. hirta*. As well, *Rickettsia* sp. has been detected in *Ctenoparia inopinata, Delostichus phyllotis, L. segnis, N. chilensis, Neotyphloceras crassispina, N. pardinasi, Neotyphloceras* sp., *N. fasciatus, Plocopsylla* sp., *S. ares, Tetrapsyllus rhombus*, and *Tetrapsyllus tantillus*, parasitizing several rodents (17). *Candidatus* Rickettsia senegalensis has been isolated around the world (92–94), but we did not find reports of human infection. Its pathogenic potential remains unknown and it has been hypothesized as a possible commensal in fleas (95). On the other hand, another *Rickettsia* sp., belonging to the spotted fever rickettsiae, has been detected in *Ornithodoros* sp., isolated from *Phyllotis darwini* (58). The pathogenicity of this rickettsia remains unknown, and it has not been isolated from the rodent host; thus, there is no evidence that it is a zoonotic rodent-borne disease. The DNA of reportedly zoonotic *Rickettsia felis* has been detected in *C. felis* from cats (96); therefore, there is no evidence that rodents participate in its reservoir. Given the above, there is not enough evidence to support zoonotic, rodent-borne rickettsioses.

Regarding relapsing fever borreliae, the DNA of the *Borrelia* sp., close to *Candidatus* Borrelia johnsonii, has been detected in *Ornithodoros* sp. isolated from *P. darwini* (58). The pathogenicity of this *Borrelia* remains unknown, and it has not been detected in rodent hosts. Therefore, there is not enough evidence to support zoonotic rodent-borne borrelioses.

The DNAs of *Candidatus* Neoehrlichia chilensis, the *Borrelia* sp. belonging to the Lyme disease group, and *Hepatozoon* sp. have been detected in *Ixodes* sp. isolated from native rodents (97). No evidence of human infection with these genotypes has been reported. Although antibodies against *Ehrlichia* sp. has been reported in humans in Chile (98), the species have not yet been determined. Additionally, *Ehrlichia canis* and *Anaplasma platys* have been detected in dogs from Chile (99, 100). Although the tick *R. sanguineus* is likely a vector (101), there is no evidence to indicate that these are rodent-borne diseases.

*Dipylidium caninum* is known to affect humans following the consumption of *Ctenocephalides* spp. or *P. irritans* (102). However, there have been no reports of *D. caninum* affecting rodents, nor fleas isolated from rodents; therefore, rodents do not seem to be part of the reservoir.

## Ecological Perspective

In all, 45.7% of generalist parasites and 6.4% of specialist parasites were shared between native and introduced rodents. Shared parasites were found in a mean of 6.9 host species, while parasites that were not shared were found in a mean of 2.1 host species. However, only fleas presented a large number of generalist species; hence, an in-depth exploration of fleas was performed. Overall, it was noted that 4.8% of the specialist (*n* = 63) and 44.8% of the generalist (*n* = 29) flea species were shared between native and introduced rodents. Consequently, the mean number of host species was 2.8 among the non-shared flea species, while it was 7.8 among the shared flea species. Considering that shared parasites must be found in at least two hosts species (both a native and an allochthonous one), it is expected that the number of hosts of shared parasites is the same number of non-shared plus one, assuming that there is no effect of the parasite's host specificity. The larger difference suggests that there is an effect of host specificity. However, this study only points toward a trend in this direction, since this hypothesis requires additional exploration to arrive at more conclusive results. Further, it should also be noted that the considered hosts must coexist in the same territory, and it was impossible to determine if that was the case in several reports.

We consider two flea species when exploring the spillover propagule pressure, *S. ares* and *N. fasciatus*, given that they were spilled over and found in many host communities (study units). Overall, we only found a significant association between the MA of *S. ares* in introduced hosts and its prevalence in native hosts (11 study units; Spearman Rho = 0.63, *P* = 0.04). A higher prevalence in the source population can be interpreted as a higher probability of contact and transmission to the recipient population. The abundance of parasites could depend on the acquisition (transmission) and reproduction of the flea within the host population. Thus, this association suggests that the higher probability of transmission of *S. ares* from the native to introduced rodents raises the acquisition by the latter. The other examined variables did not show significant associations. The lack of an association suggests that the parasites already circulate within the recipient population independently of the source population. This is only a preliminary exploration, as this analysis does not consider the sample size nor the variability within each study unit. A previous study only found an association between loads in the source population and the fact that the parasite was transmitted (13).

## Concluding Remarks

Although there are many reports of vector-borne pathogens, most of them lack sufficient evidence to state they are rodent-borne zoonoses. Thus, most of the rodent-borne zoonoses reviewed herein represent cases of parasitosis transmitted by invasive rodents, enhancing their importance for human health. Therefore, in the case of vector-borne zoonoses, it is necessary to remain focused on searching for the pathogens on rodents, while also describing the entire cycle to determine whether they are, in fact, rodent-borne zoonoses. Specifically, it is important to determine the presence of these pathogens in humans, rodents, and vectors, while also isolating the vector from both humans and rodents. This will enhance our awareness of the risks rodents carry.

The rodent-borne helminthiases have decreased in Chile over time (26, 75); however, it is necessary to keep vigilant. Most zoonotic helminths of rodents are carried by introduced rodents, in such a way that either introduced rodents are of greater concern for humans than are native rodents, or it may be the case that many instances of parasitosis have been caused by native parasites, but were misidentified as an introduced parasite. For instance, an undetermined *Hymenolepis* (*Rodentolepis*) species was found only in cricetid rodents in the presence of rats and mice, suggesting that this is a native parasite (13). Thus, *H*. (*Rodentolepis*) *nana* eggs reported in humans could have been misidentified, corresponding to this native cestode. The introduction of molecular tools could help to corroborate this identification in order to determine the potential parasite source.

Few studies have examined the sharing of parasites between native and introduced rodents. Although some factors involved in this transmission have been identified or observed, the consequences of allochthonous parasites on native host and parasite populations and individuals have not been studied. Although there is no evidence to support the notion that host–guest associations are related to parasitism, some pathological studies have shown the extent of damage to the hosts, supporting the idea that they are parasites. For instance, *O. bacoti, Plocopsylla* sp., *Heterakis spumosa*, and *H. taeniaeformis* produced lesions in rats (103). Further, biological evidence obtained from guests supports the notion that they are parasites, as they are hematophagous. For instance, although not all *Androlaelaps* spp. are obligate parasites, *A. farenholzi* is reportedly unable to reproduce on a diet of arthropods alone (predation), instead requiring blood or a mixed diet (104). No studies to date have been conducted to examine the effects of parasites on host populations in Chile. Rather, these effects on individual hosts are the only evidence to suggest that introduced parasites can affect native hosts by parasite transmission. From the point of view of biological conservation, this is an important issue to assess in future studies.

## Author Contributions

LMS, MS, DG-A, and AH reviewed the literature and provided the raw data. CL-A performed the analysis of the literature and data, and wrote the first draft. All authors revised and expanded upon the original draft and approved of the submitted manuscript.

## Conflict of Interest

The authors declare that the research was conducted in the absence of any commercial or financial relationships that could be construed as a potential conflict of interest.
