# Supplementary material for: Parasites of Native and Invasive Rodents in Chile: Ecological and Human Health Needs
Source: Front Vet Sci. 2021 Feb 11;8:643742. doi: 10.3389/fvets.2021.643742 (PMC7905021; doi:10.3389/fvets.2021.643742)
Supplement: Supplementary file 1 [file Data_Sheet_1.docx]

Supplementary Material

# Supplementary table 1: List of helminth parasites of rodents in Chile and their hosts. Cites and references follow the order of the main text.

| **Parasite** | **Allochthonous host** | **Native host** | **Reference** |
| --- | --- | --- | --- |
| **TREMATODA** |  |  |  |
| **Plagiorchiida** |  |  |  |
| *Hippocrepis myocastoris* |  | *Myocastor copypus* | (18) |
| **CESTODA** | | | |
| **Cyclophyllidea** | | | |
| *Andrya octodonensis* |  | *Octodon degus* | (13, 19) |
|  |  | *Phyllotis darwini* |  |
| *Diplophallus taglei* |  | *Lagidium peruanum* | (20) |
| *Echinococcus granulosus* |  | *Octodon degus* | (21) |
| *Hymenolepis diminuta* | *Rattus rattus* |  | (13) |
|  | *Rattus norvegicus* |  |  |
| *Hymenolepis nana* | *Rattus* sp. |  | (22) |
| *Hymenolepis microstoma* | *Rattus* sp. |  | (22) |
| *Hydatigera taeniformis* (syn. *Taenia taeniaeformis*, *T. crassicollis*) | *Rattus norvegicus* | *Phyllotis xanthopygus* | (13, 23) |
| *Monoecocestus torresi* |  | *Ctenomys maulinus* | (24) |
| **NEMATODA** | | | |
| **Trichinellida/Trichocephalida** | | | |
| *Calodium hepaticum* | *Rattus norvegicus* |  | (25) |
| *Anatrichosoma* sp. |  | *Abrothrix olivacea* | (13) |
| *Trichinella spiralis* | *Rattus norvegicus* |  | (26) |
| *Trichosomoides crassicauda* | *Rattus norvegicus* |  | (25) |
| *Trichuris bradleyi* |  | *Octodon degus* | (13, 27) |
| *Trichuris chilensis* |  | *Abrothrix longipilis* | (28) |
| *Trichuris fulvis* |  | *Ctenomys fulvus* | (29) |
| *Trichuris muris* | *Mus musculus* |  | (13) |
| *Trichuris myocastoris* |  | *Myocastor coypus* | (18) |
| *Trichuris pardinasi* |  | *Phyllotis darwini* | (13) |
| *Trichuris robustis* |  | *Ctenomys fulvus* | (30) |
| **Rhabditida** |  |  |  |
| *Strongyloides ratti* | *Rattus* sp. |  | (22) |
| **Strongylida** | | | |
| *Graphidioides myocastorii* |  | *Myocastor coypus* | (18) |
| *Graphidioides taglei* |  | *Octodon degus* | (19) |
| *Graphidioides yanezi* |  | *Spalacopus cyanus* | (31) |
| *Inglamidium akodon* |  | *Abrothrix olivacea* | (32) |
| *Nippostrongylus brasiliensis* | *Rattus* *rattus* |  | (13, 25) |
|  | *Rattus norvegicus* |  |  |
| *Pudica cattani* |  | *Octodon degus* | (13, 33) |
| *Pudica degusi* |  | *Octodon degus* | (13, 33) |
| *Stilestrongylus manni* |  | *Abrothrix olivacea* | (34) |
|  |  | *Abrothrix longipilis* |  |
|  |  | *Oligoryzomys longicaudatus* |  |
| *Stilestrongylus valdivianus* |  | *Loxodontomys micropus* | (35) |
| **Ascaridida** | | | |
| *Heterakis spumosa* | *Mus musculus* | *Abrothrix olivacea* | (13, 25, 36, 37) |
|  | *Rattus rattus* |  |  |
|  | *Rattus norvegicus* |  |  |
| **Spirurida** | | | |
| *Dipetalonema travassosi* |  | *Myocastor coypus* | (18) |
| *Gongylonema neoplasticum* | *Rattus* sp. |  | (22) |
| *Litomosoides pardinasi* | *Rattus rattus* | *Oligoryzomys longicaudatus* | (13, 38) |
|  |  | *Phyllotis darwini* |  |
| *Paraspidodera uncinata* |  | *Ctenomys maulinus* | (25) |
| *Physaloptera calnuensis* | *Mus musculus* | *Abrothrix olivacea* | (13, 37, 39) |
|  | *Rattus rattus* | *Oligoryzomys longicaudatus* |  |
|  | *Rattus norvegicus* | *Octodon degus* |  |
| *Protospirura numidicola* |  | *Abrothrix longipilis* | (13) |
| *Rictularia* sp. | *Rattus norvegicus* |  | (25) |
| **Oxyurida** | | | |
| *Aspiculuris tetraptera* | *Mus musculus* |  | (13, 39) |
|  | *Rattus rattus* |  |  |
| *Helminthoxys gigantea* |  | *Octodon degus* | (40) |
| *Heteroxinema chilensis* |  | *Octodon degus* | (41) |
| *Syphacia obvelata* | *Mus musculus* | *Abrothrix olivacea* | (13, 37, 39) |
| *Syphacia muris* | *Rattus rattus* | *Phyllotis darwini* | (13, 42) |
|  | *Rattus norvegicus* |  |  |
| *Syphacia phyllotios* |  | *Phyllotis darwini* | (13, 43, 44) |

# Supplementary Table 2: List of arthropod parasites of rodents in Chile and their hosts. Cites and references follow the order of the main text.

| **Parasite** | **Allochthonous host** | **Native host** | **Reference** |
| --- | --- | --- | --- |
| **ARACHNIDA** | | | |
| **Mesostigmata** | | | |
| *Androlaelaps farenholzi* |  | *Abrocoma bennetti* | (16, 45, 46) |
|  |  | *Abrothrix olivacea* |  |
|  |  | *Phyllotis xanthopygus* |  |
|  |  | *Akodon albiventer* |  |
| *Gigantolaelaps wolffsohni* |  | *Oligoryzomys longicaudatus* | (47) |
| *Laelaps echidninus* | *Rattus rattus* |  | (47) |
|  | *Rattus norvegicus* |  |  |
| *Lukoschus maresi* |  | *Abrothrix olivacea* | (47) |
| *Mysolaelaps microspinosus* |  | *Oligoryzomys longicaudatus* | (47) |
| *Ornithonyssus* sp*.* | *Rattus rattus* | *Abrocoma bennetti* | (45, 46, 48, 49) |
|  |  | *Abrothrix olivacea* |  |
|  |  | *Abrothrix hirta* |  |
|  |  | *Abrothrix longipilis* |  |
|  |  | *Abrothrix sanborni* |  |
|  |  | *Loxodontomys micropus* |  |
|  |  | *Chelemys macronyx* |  |
|  |  | *Phyllotis darwini* |  |
| **Trombidiformes** | | | |
| *Akodonacarus martini* |  | “*Akodon* sp.” (probably*Abrothrix* sp.) | (50) |
| *Chilacarus martini* |  | *Abrothrix hirta* | (51) |
| *Dewacarus lemuensis* |  | *Loxodontomys pikumche* | (52) |
| *Herpetacarus eloisae* |  | *Abrothrix olivacea* | (53) |
|  |  | *Abrothrix sanborni* |  |
|  |  | *Geoxus valdivianus* |  |
| *Paratrombicula goffi* |  | *Abrothrix olivacea* | (53) |
| *Poliremotus chilensis* |  | *Octodon degus* | (54) |
| *Paraguacarus santiagoensis* |  | *Octodon degus* | (55) |
| *Quadraseta chiloensis* |  | *Abrothrix olivacea* | (53) |
|  |  | *Geoxus valdivianus* |  |
|  |  | *Oligoryzomys longicaudatus* |  |
| **Sarcoptiformes** | | | |
| *Notoedres muris (syn. N. alepis)* | *Rattus* sp*.* |  | (22) |
| *Amlistrophorus geoxus* |  | *Geoxus valdivianus* | (56) |
| **Ixodida** | | | |
| *Ixodes abrocomae* |  | *Abrocoma bennetti* | (57) |
|  |  | *Abrothrix longipilis* |  |
|  |  | *Abrothrix olivacea* |  |
|  |  | *Phyllotis xanthopygus* |  |
| *Ixodes sigelos* | *Rattus norvegicus* | *Aconaemys fuscus* | (14) |
|  |  | *Phyllotis* sp. |  |
|  |  | *Abrocoma bennetti* |  |
|  |  | *Octodon degus* |  |
|  |  | *Oligoryzomys longicaudatus* |  |
| *Ornithodoros* sp. Cachapoal |  | *Phyllotis darwini* | (58) |
| *Ornithodoros octodontus* |  | *Octodon degus* | (59) |
| *Rhipicephalus sanguineus* | *Rattus norvegicus* |  | (14) |
| **INSECTA** | | | |
| **Phthiraptera** | | | |
| *Abrocomophaga chilensis* |  | *Abrocoma bennetti* | (46, 60) |
| *Abrocomophaga hellenthali* |  | *Octodon degus* | (61) |
| *Abrocomaphthirus chilensis* |  | *Abrocoma bennetti* | (62, 63) |
| *Ferrisella chilensis* |  | *Octodon degus* | (64) |
| *Ferrisella disgrega* |  | *Octodontomys gliroides* | (64) |
| *Gyropus elongatus* |  | *Aconaemys fuscus* | (64) |
| *Gyropus distinctus* |  | *Abrocoma bennetti* | (46, 64, 65) |
|  |  | *Octodon degus* |  |
|  |  | *Octodon lunatus* |  |
| *Gyropus parvus* |  | *Ctenomys maulinus* | (64) |
| *Gyropus latipllicaris* |  | *Ctenomys osgoodi* | (66) |
| *Hoplopleura travassosi* |  | *Oligoryzomys longicaudatus* | (67, 68) |
| *Hoplopleura andina* |  | *Abrothrix olivacea* | (63, 67-69) |
|  |  | *Abrothrix andinus* |  |
|  |  | *Geoxus valdivianus* |  |
| *Hoplopleura pacifica* | *Rattus norvegicus* |  | (67) |
| *Hoplopleura aitkeni* |  | *Phyllotis darwini* | (67) |
|  |  | *Phyllotis xanthopygus* |  |
| *Monogyropus longus* |  | *Abrocoma bennetti* | (46, 66) |
| *Phtheiropoios pearsoni* |  | *Abrocoma bennetti* | (64) |
| *Phtheiropoios pollicaris* |  | *Ctenomys magellanicus* | (64) |
| *Phtheiropoios nematophallus* |  | *Ctenomys opimus* | (64) |
|  |  | *Phyllotis xanthopygus* |  |
| **Siphonaptera** | | | |
| *Agastopsylla boxi* |  | *Loxodontomys micropus* | (15) |
|  |  | *Abrothrix olivacea* |  |
|  |  | *Abrothrix longipilis* |  |
|  |  | *Abrothrix xanthorhinus* |  |
|  |  | *Reithrodon physodes* |  |
|  |  | *Phyllotis xanthopygus* |  |
|  |  | *Euneomys mordax* |  |
| *Agastopsylla hirsutior* |  | *Abrothrix longipilis* | (15) |
| *Agastopsylla guzmani* |  | *Akodon albiventer* | (15) |
| *Agastopsylla nylota* |  | *Euneomys chinchilloides* | (15) |
| *Agastopsylla pearsoni* |  | *Abrothrix longipilis* | (15) |
| *Barreropsylla excelsa* |  | *Abrothrix longipilis* | (15) |
|  |  | *Abrothrix olivacea* |  |
|  |  | *Loxodontomys micropus* |  |
|  |  | *Geoxus valdivianus* |  |
|  |  | *Oligoryzomys longicaudatus* |  |
| *Chiliopsylla allophyla* |  | *Abrothrix longipilis* | (15) |
|  |  | *Abrothrix olivacea* |  |
|  |  | *Abrothrix sanborni* |  |
|  |  | *Abrothrix brachiotis* |  |
|  |  | *Oligoryzomys longicaudatus* |  |
|  |  | *Loxodontomys micropus* |  |
|  |  | *Chelemys macronyx* |  |
|  |  | *Irenomys tarsalis* |  |
| *Cleopsylla townsendi* |  | *Akodon* sp. | (15) |
|  |  | *Octodon lunatus* |  |
| *Craneopsylla minerva* |  | *Phyllotis darwini* | (15) |
|  |  | *Phyllotis xanthopygus* |  |
|  |  | *Abrothrix olivacea* |  |
|  |  | *Reithrodon physodes* |  |
| *Ctenocephalides canis* | *Rattus norvegicus* |  | (22) |
| *Ctenocephalides felis* | *Rattus rattus* |  | (15, 22) |
|  | *Rattus norvegicus* |  |  |
| *Ctenoparia inopinata* | *Rattus rattus* | *Abrothrix brachiotis* | (15) |
|  |  | *Abrothrix longipilis* |  |
|  |  | *Abrothrix olivacea* |  |
|  |  | *Abrothrix sanborni* |  |
|  |  | *Geoxis valdivianus* |  |
|  |  | *Oligorizomys longicaudatus* |  |
|  |  | *Phyllotis darwini* |  |
|  |  | *Loxodontomys micropus* |  |
|  |  | *Aconaemys porteri* |  |
| *Ctenoparia intermedia* |  | *Reithrodon s*p. | (15) |
|  |  | *Loxodontomys micropus* |  |
| *Ctenoparia jordani* | *Rattus rattus* | *Abrothrix hirta* | (12, 15, 17) |
|  |  | *Abrothrix olivacea* |  |
|  |  | *Loxodontomys micropus* |  |
|  |  | *Oligoryzomys longicaudatus* |  |
| *Ctenoparia topali* |  | *Abrothrix brachiotis* | (15) |
|  |  | *Abrothrix longipilis* |  |
|  |  | *Abrothrix olivacea* |  |
|  |  | *Abrothrix sanborni* |  |
|  |  | *Loxodontomys micropus* |  |
|  |  | *Oligorizomys longicaudatus* |  |
|  |  | *Spalacopus cyanus* |  |
| *Dasypsyllus aedon* |  | *Abrothrix longipilis* | (15) |
| *Dasypsyllus araucanus* |  | *Abrothrix olivacea* | (15) |
| *Delostichus coxalis* | *Rattus rattus* | *Abrothrix olivacea* | (15) |
|  |  | *Octodon degus* |  |
|  |  | *Octodon lunatus* |  |
|  |  | *Abrocoma bennetti* |  |
| *Delostichus degus* |  | *Octodon degus* | (15) |
|  |  | *Abrocoma bennetti* |  |
| *Delostichus octomyos* |  | *Octomys mimax* | (15) |
|  |  | *Octodon degus* |  |
| *Delostichus phyllotis* |  | *Octodon degus* | (15, 17) |
|  |  | *Octodon bridgesi* |  |
|  |  | *Abrocoma bennetti* |  |
|  |  | *Phyllotis darwini* |  |
| *Delostichus smiti* | *Rattus rattus* | *Phyllotis darwini* | (15, 46) |
|  |  | *Abrothrix longipilis* |  |
|  |  | *Octodon degus* |  |
|  |  | *Abrocoma bennetti* |  |
| *Echidnophaga gallinacea* | *Rattus norvegicus* | *Cavia porcellus* | (15) |
| *Ectinorus angularis* |  | *Abrothrix olivacea* | (15) |
|  |  | *Akodon* sp. |  |
| *Ectinorus chilensis* |  | *Oligoryzomys longicaudatus* | (15, 17) |
|  |  | *Abrocoma bennetti* |  |
|  |  | *Aconaemys fuscus* |  |
|  |  | *Octodon degus* |  |
|  |  | *Spalacopus cyanus* |  |
| *Ectinorus cocyti* |  | *Abrothrix olivacea* | (15, 17) |
|  |  | *Octodon degus* |  |
|  |  | *Spalacopus cyanus* |  |
|  |  | *Abrocoma bennetti* |  |
| *Ectinorus curvatus* |  | *Phyllotis darwini* | (15) |
| *Ectinorus deplexus* |  | *Euneomys chinchilloides* | (15) |
| *Ectinorus gallardoi* |  | *Aconaemys fuscus* | (15) |
| *Ectinorus hertigi* |  | *Ctenomys fulvus* | (15) |
| *Ectinorus ineptus* |  | *Phyllotis darwini* | (15) |
|  |  | *Eligmodontia puerulus* |  |
| *Ectinorus insignis* |  | *Eligmodontia puerulus* | (15) |
| *Ectinorus ixanus* |  | *Loxodontomys micropus* | (15) |
| *Ectinorus lagidium* |  | *Lagidium viscacia* | (15) |
| *Ectinorus levipes* |  | *Abrothrix longipilis* | (15) |
| *Ectinorus martini* |  | *Aconaemys fuscus* | (15) |
|  |  | *Aconaemys porteri* |  |
|  |  | *Aconaemys sagei* |  |
|  |  | *Geoxus valdivianus* |  |
|  |  | *Chelemys macronyx* |  |
|  |  | *Abrothrix olivacea* |  |
| *Ectinorus mondacai* |  | *Aconaemys fuscus* | (15) |
|  |  | *Aconaemys porteri* |  |
| *Ectinorus nomisis* |  | *Octodontomys gliroides* | (15) |
|  |  | *Andinomys edax* |  |
| *Ectinorus onychius* |  | *Abrothrix longipilis* | (15) |
|  |  | *Abrothrix olivacea* |  |
|  |  | *Abrothrix xanthorhinus* |  |
|  |  | *Loxodontomys micropus* |  |
|  |  | *Reithrodon physodes* |  |
|  |  | *Euneomys* sp. |  |
|  |  | *Phyllotis xanthopygus* |  |
|  |  | *Eligmodontia typus* |  |
| *Ectinorus pilosus* |  | *Ctenomys* sp. | (15) |
| *Ectinorus setosicornis* |  | *Lagidium viscacia* | (15) |
| *Ectinorus simonsi* |  | *Octodontomys gliroides* | (15) |
| *Ectinorus splendidus* |  | *Euneomys chinchilloides* | (15) |
| *Ectinorus uncinatus* |  | *Andinomys edax* | (15) |
|  |  | *Abrothrix* sp. |  |
| *Hectopsylla cypha* |  | *Abrothrix longipilis* | (15) |
|  |  | *Abrothrix olivacea* |  |
|  |  | *Oligoryzomys longicaudatus* |  |
|  |  | *Phyllotis darwini* |  |
|  |  | *Phyllotis xanthopygus* |  |
| *Hectopsylla gemina* |  | *Abrocoma benetti* | (15) |
|  |  | *Phyllotis darwini* |  |
| *Hectopsylla suarezi* | *Rattus rattus* | *Ctenomys robustus* | (15) |
|  | *Rattus norvegicus* | *Octodontomys gliroides* |  |
| *Leptopsylla segnis* | *Rattus rattus* | *Abrothrix olivacea* | (15, 17) |
|  | *Rattus norvegicus* | *Oligoryzomys longicaudatus* |  |
|  |  | *Octodon degus* |  |
| *Listronius fortis* |  | *Abrothrix longipilis* | (15) |
|  |  | *Abrothrix olivacea* |  |
| *Listronius plesiomorphus* |  | *Abrothrix longipilis* | (15) |
|  |  | *Chelemys macronyx* |  |
| *Listronius ulus* |  | *Abrothrix longipilis* | (15) |
|  |  | *Abrothrix olivacea* |  |
|  |  | *Loxodontomys micropus* |  |
|  |  | *Reithrodon physodes* |  |
| *Neotyphloceras crassispina* | *Rattus rattus* | *Abrothrix longipilis* | (15) |
|  |  | *Abrothrix olivacea* |  |
|  |  | *Abrothrix andinus* |  |
|  |  | *Akodon albiventer* |  |
|  |  | *Akodon berlepschii* |  |
|  |  | *Loxodontomys micropus* |  |
|  |  | *Oligoryzomys longicaudatus* |  |
|  |  | *Phyllotis darwini* |  |
|  |  | *Phyllotis xanthopygus* |  |
|  |  | *Chelemys macronyx* |  |
|  |  | *Reithrodon physodes* |  |
|  |  | *Eligmodontia* sp. |  |
|  |  | *Abrocoma cinerea* |  |
|  |  | *Ctenomys opimus* |  |
|  |  | *Aconaemys porteri* |  |
|  |  | *Octodontomys gliroides* |  |
|  |  | *Octodon degus* |  |
| *Neotyphloceras chilensis* | *Rattus rattus* | *Abrothrix longipilis* | (15) |
|  |  | *Abrothrix olivacea* |  |
|  |  | *Abrothrix xanthorhinus* |  |
|  |  | *Phyllotis darwini* |  |
|  |  | *Phyllotis xanthopygus* |  |
|  |  | *Oligoryzomys longicaudatus* |  |
|  |  | *Reithrodon* sp. |  |
|  |  | *Octodon degus* |  |
|  |  | *Octodon bridgesi* |  |
|  |  | *Aconaemys* sp. |  |
|  |  | *Abrocoma bennetti* |  |
| *Neotyphloceras pardinasi* | *Rattus rattus* | *Abrothrix olivacea* | (12, 17) |
|  |  | *Abrothrix hirta* |  |
|  |  | *Oligoryzomys longicaudatus* |  |
| *Nonnapsylla rothschildi* |  | *Octodontomys gliroides* | (15) |
|  |  | *Abrocoma cinerea* |  |
| *Nosopsyllus fasciatus* | *Rattus rattus* | *Abrothrix hirta* | (15, 17) |
|  |  | *Oligoryzomys longicaudatus* |  |
|  |  | *Abrothrix olivacea* |  |
|  |  | *Aconaemys porteri* |  |
|  |  | *Myocastor coypus* |  |
| *Plocopsylla achilles* | *Rattus rattus* | *Abrothrix longipilis* | (15) |
| *Plocopsylla consobrina* |  | *Abrothrix longipilis* | (15) |
| *Plocopsylla crypta* |  | *Abrothrix olivacea* | (15) |
|  |  | *Phyllotis darwini* |  |
|  |  | *Octodon degus* |  |
| *Plocopsylla diana* |  | *Abrothrix longipilis* | (15) |
|  |  | *Abrothrix olivacea* |  |
|  |  | *Aconaemys porteri* |  |
| *Plocopsylla enderleini* |  | *Chinchillula sahamae* | (15) |
| *Plocopsylla fuegina* |  | *Ctenomys magellanicus* | (15) |
| *Plocopsylla lewisi* |  | *Abrothrix longipilis* | (15) |
|  |  | *Abrothrix olivacea* |  |
|  |  | *Oligoryzomys longicaudatus* |  |
|  |  | *Reithrodon physodes* |  |
|  |  | *Euneomys chinchilloides* |  |
| *Plocopsylla muruai* |  | *Abrothrix olivacea* | (15) |
|  |  | *Abrothrix longipilis* |  |
| *Plocopsylla reigi* |  | *Abrothrix olivacea* | (15) |
|  |  | *Oligoryzomys longicaudatus* |  |
|  |  | *Phyllotis darwini* |  |
|  |  | *Geoxus valdivianus* |  |
|  |  | *Chelemys macronyx* |  |
| *Plocopsylla silewi* |  | *Abrothrix xanthorhinus* | (15) |
| *Plocopsylla viracocha* |  | *Auliscomys boliviensis* | (15) |
|  |  | *Andinomys edax* |  |
|  |  | *Phyllotis darwini* |  |
|  |  | *Chinchillula sahamae* |  |
|  |  | *Phyllotis osgoodi* |  |
| *Plocopsylla wilesi* |  | *Abrothrix xanthorhinus* | (15) |
|  |  | *Phyllotis xanthorhinus* |  |
| *Plocopsylla wolffsohni* | *Rattus rattus* | *Abrothrix longipilis* | (12, 15, 17) |
|  |  | *Abrothrix olivacea* |  |
|  |  | *Chelemys macronyx* |  |
|  |  | *Phyllotis darwini* |  |
|  |  | *Octodon degus* |  |
| *Polygenis platensis* |  | *Ctenomys robustus* | (15) |
|  |  | *Ctenomys fulvus* |  |
| *Pulex irritans* | *Mus musculus* | *Lagidium viscacia* | (22, 70) |
|  | *Rattus rattus* |  |  |
|  | *Rattus norvegicus* |  |  |
| *Sphinctopsylla ares* | *Rattus rattus* | *Abrothrix lanosus* | (12, 15, 17) |
|  |  | *Abrothrix longipilis* |  |
|  |  | *Abrothrix olivacea* |  |
|  |  | *Abrothrix sanborni* |  |
|  |  | *Abrothrix xanthorhinus* |  |
|  |  | *Oligoryzomys longicaudatus* |  |
|  |  | *Geoxus valdivianus* |  |
|  |  | *Chelemys macronyx* |  |
|  |  | *Loxodontomys micropus* |  |
|  |  | *Reithrodon physodes* |  |
|  |  | *Euneomys mordax* |  |
|  |  | *Loxodontomys pikumche* |  |
|  |  | *Phyllotis darwini* |  |
|  |  | *Phyllotis xanthopygus* |  |
|  |  | *Spalacopus cyanus* |  |
|  |  | *Aconaemys porteri* |  |
| *Tetrapsyllus amplus* |  | *Oligoryzomys longicaudatus* | (15, 17) |
|  |  | *Abrothrix olivacea* |  |
|  |  | *Abrothrix longipilis* |  |
|  |  | *Phyllotis darwini* |  |
| *Tetrapsyllus bleptus* |  | *Abrothrix* sp. | (15) |
|  |  | *Phyllotis darwini* |  |
|  |  | *Phyllotis magister* |  |
|  |  | *Phyllotis xanthophygus* |  |
| *Tetrapsyllus comis* |  | *Octodon bridgesi* | (15) |
| *Tetrapsyllus contortrix* |  | *Abrothrix olivacea* | (15) |
|  |  | *Phyllotis darwini* |  |
| *Tetrapsyllus corfidii* |  | *Abrothrix longipilis* | (15, 17) |
|  |  | *Abrothrix olivacea* |  |
|  |  | *Abrothrix olivacea* |  |
|  |  | *Phyllotis darwini* |  |
|  |  | *Aconaemys fuscus* |  |
|  |  | *Octodon degus* |  |
|  |  | *Octodon bridgesi* |  |
|  |  | *Octodon lunatus* |  |
|  |  | *Abrocoma bennetti* |  |
| *Tetrapsyllus elutus* |  | *Auliscomys sublimis* | (15) |
|  |  | *Phyllotis xanthopygus* |  |
| *Tetrapsyllus maulinus* |  | *Ctenomys maulinus* | (15) |
|  |  | *Ctenomys magellanicus* |  |
|  |  | *Ctenomys colburni* |  |
| *Tetrapsyllus rhombus* | *Rattus rattus* | *Abrothrix olivacea* | (12, 15, 17) |
|  | *Rattus norvegicus* | *Abrothrix longipilis* |  |
|  |  | *Abrothrix brachiotis* |  |
|  |  | *Loxodontomys micropus* |  |
|  |  | *Euneomys mordax* |  |
|  |  | *Geoxus valdivianus* |  |
|  |  | *Oligoryzomys longicaudatus* |  |
|  |  | *Reithrodon physodes* |  |
|  |  | *Phyllotis darwini* |  |
|  |  | *Loxodontomys pikumche* |  |
|  |  | *Aconaemys porteri* |  |
|  |  | *Ctenomys maulinus* |  |
| *Tetrapsyllus satyrus* |  | *Aconaemys fuscus* | (15) |
|  |  | *Aconaemys porteri* |  |
|  |  | *Chelemys macronyx* |  |
|  |  | *Geoxus valdivianus* |  |
| *Tetrapsyllus simulans* |  | *Abrothrix longipilis* | (15) |
|  |  | *Oligoryzomys longicaudatus* |  |
|  |  | *Phyllotis darwini* |  |
| *Tetrapsyllus tantillus* |  | *Abrothrix olivacea* | (15, 17) |
|  |  | *Abrothrix longipilis* |  |
|  |  | *Abrothrix xanthorhinus* |  |
|  |  | *Euneomys mordax* |  |
|  |  | *Reithrodon physodes* |  |
|  |  | *Loxodontomys micropus* |  |
|  |  | *Phyllotis darwini* |  |
|  |  | *Phyllotis xanthopygus* |  |
|  |  | *Spalacopus cyanus* |  |
|  |  | *Ctenomys magellanicus* |  |
|  |  | *Octodon degus* |  |
|  |  | *Abrocoma bennetti* |  |
| *Tiamastus callens* |  | *Ctenomys maulinus* | (15) |
| *Tiamastus cavicola* |  | *Cavia* sp. | (15) |
| *Tiamastus gallardoi* |  | *Abrothrix olivacea* | (15) |
|  |  | *Ctenomys colburni* |  |
| *Tiamastus plesius* |  | *Ctenomys robustus* | (15) |
| *Tunga bonneti* | *Rattus rattus* | *Phyllotis darwini* | (15) |
|  |  | *Phyllotis xanthopygus* |  |
| *Tunga libis* |  | *Phyllotis darwini* | (15) |
| *Tunga penetrans* | *Rattus norvegicus* |  | (15) |
| *Xenopsylla astia* | *Rattus norvegicus* |  | (15) |
| *Xenopsylla cheopis* | *Rattus rattus* |  | (15) |
|  | *Rattus norvegicus* |  |  |

# References cited in supplementary tables

12. Moreno Salas L, Espinoza-Carniglia M, Lizama Schmeisser N, Torres LG, Silva-de la Fuente MC, Lareschi M, et al. Fleas of black rats (*Rattus rattus*) as reservoir host of *Bartonella* spp. in Chile. *PeerJ.* (2019) 7**:** e7371. doi: 10.7717/peerj.7371

13. Landaeta-Aqueveque C, Robles MdR, Henríquez A, Yáñez-Meza A, Correa JP, González-Acuña D, et al. Phylogenetic and ecological factors affecting the sharing of helminths between native and introduced rodents in Central Chile. *Parasitology.* (2018) 145**:** 1570-6. doi: 10.1017/S0031182018000446

14. González-Acuña D, Guglielmone A. Ticks (Acari: Ixodoidea: Argasidae, Ixodidae) of Chile. *Experimental & Applied Acarology.* (2005) 35**:** 147-63. doi: 10.1007/s10493-004-1988-2

15. Beaucournu J-C, Moreno L, González-Acuña D. Fleas (Insecta-Siphonaptera) of Chile: a review. *Zootaxa.* (2014) 3900**:** 151-203. doi: 10.11646/zootaxa.3900.2.1

16. Silva-de la Fuente MC, Moreno Salas L, Casanueva ME, Lareschi M, González-Acuña D. Morphometric variation of *Androlaelaps fahrenholzi* (Mesostigmata: Laelapidae) associated with three Sigmodontinae (Rodentia: Cricetidae) from the north of Chile. *Experimental and Applied Acarology.* (2020) 81**:** 135-48. doi: 10.1007/s10493-020-00490-6

17. Moreno-Salas L, Espinoza-Carniglia M, Lizama-Schmeisser N, Torres-Fuentes LG, Silva-de La Fuente MC, Lareschi M, et al. Molecular detection of *Rickettsia* in fleas from micromammals in Chile. *Parasites & Vectors.* (2020) 13**:** 523. doi: 10.1186/s13071-020-04388-5

18. Babero BB, Cabello C, Kinard JE. Helmintofauna de Chile. V. Parásitos del Coipo, *Myocastor coypus* (Molina, 1782). *Boletin Chileno de parasitologia.* (1979) 34**:** 26-31.

19. Babero BB, Cattan PE. Helmintofauna de Chile: III. Parasitos del roedor degu, *Octodon degus* Molina, 1782, con la descripcion de tres nuevas especies. *Boletin Chileno de Parasitologia.* (1975) 30**:** 68-76.

20. Olsen W. *Diplophallus taglei* n. sp. (Cestoda: Cyclophyllidea) from the Viscacha, *Lagidium peruanum* Meyer, 1832 (Chinchillidae) from the Chilean Andes. *Proceedings of the Helminthological Society of Washington.* (1966) 33**:** 49-53.

21. Álvarez V. Investigaciones sobre equinococosis silvestre en Chile. *Biológica.* (1961) 31**:** 89-94.

22. Ruiz del Río A. Contribución al estudio de las enfermedades parasitarias humanas transmitidas por las ratas en Concepción. *Boletín de la Sociedad de Biología de Concepción.* (1939) 13**:** 47-82.

23. Cubillos V, Torres P, Gallardo M. Aspectos histopatológicos en un nuevo hospedador de *Taenia taeniformis*. *Archivos de Medicina Veterinaria.* (1991) 33**:** 77-9.

24. Olsen OW. *Monocoestus torresi* n. sp. Cestoda: Cyclophyllidea: Anoplocephalidae from tuco-tuco, *Ctenomys maulinus* brunneus Osgood 1943. *Revista Ibérica de Parasitología.* (1976) 38**:** 505-14.

25. Torres P, Lopetegui O, Gallardo M. Investigaciones sobre algunos nematodos parásitos de *Rattus norvegicus* y *Ctenomys maulinus* de Chile. *Boletín Chileno de Parasitología.* (1976) 31**:** 39-42.

26. Schenone H, Olea A, Schenone H, Contreras M, Mercado R, Sandoval L, et al. Situación epidemiológica actual de la triquinosis en Chile. 1991-2000. *Revista Médica de Chile.* (2002) 130**:** 281-5. doi: DOI: 10.4067/S0034-98872002000300006

27. Babero BB, Cattan PE, Cabello C. *Trichuris bradleyi* sp. N., a whipworm from *Octodon degus* in Chile. *The Journal of Parasitology.* (1975) 61**:** 1061-3.

28. Babero BB, Cattan PE, Cabello C. A new species of whipworm from the rodent *Akodon longipilis* in Chile. *Transactions of the American Microscopical Society.* (1976) 95**:** 232-5.

29. Babero BB, Murua R. The Helminth Fauna of Chile. X. A New Species of Whipworm from a Chilean Rodent. *Transactions of the American Microscopical Society.* (1987) 106**:** 190-3. doi: 10.2307/3226320

30. Babero BB, Murua RB. A new species of whipworm from a south american hystricomorph rodent. *Memórias do Instituto Oswaldo Cruz.* (1990) 85**:** 211-3. doi: 10.1590/s0074-02761990000200012

31. Babero BB, Cattan PE. Helmintofauna de Chile: VIII. *Graphioides yañezi* sp. n. Parásito de *Spalacopus cyanus* Molina. *Boletìn del Museo Nacional de Historia Natural de Chile.* (1980) 37**:** 225-8

32. Durette-Desset M-C, Denke M. Présence chez un rongeur du Chili d’un nématode Inglamidinae (sub. fam. nov.) appartenant aux Amidostomatidae, famille connue des mammifères d’Australie. *Annales de Parasitologie Humaine et Comparée.* (1976) 51**:** 453-60. doi: 10.1051/parasite/1976514453

33. Digiani MC, Landaeta-Aqueveque C, Serrano PC, Notarnicola J. Pudicinae (Nematoda: Heligmonellidae) Parasitic in Endemic Chilean Rodents (Caviomorpha: Octodontidae and Abrocomidae): Description of a New Species and Emended Description of *Pudica degusi* (Babero and Cattan) n. comb. *Journal of Parasitology.* (2017) 103**:** 736-46. doi: 10.1645/17-81

34. Denke MA, Murúa R. Description de *Stilestrongylus manni* n. sp. (Nematoda: Heligmosomidae) parasite de différents Cricétidés du Chili. *Bulletin du Muséum National D’Histoire Naturelle 3 série, n° 428.* (1977) Zoologie 298**:** 127-31.

35. Durette-Desset M-C, Murua R. Description de *Stilestrongylus valdivianus* n. sp. (Nematoda, Heligmonellidae), parasite d'un Cricétidé du Chili. *Bulletin du Muséum National D’Histoire Naturelle 4 série, n° 1.* (1979) Section A, n°1**:** 245-9.

36. Palomino H, Barriga OO. *Heterakis spumosa* en rata sinantrópica. *Boletin Chileno de Parasitologia.* (1967) 22**:** 79.

37. Landaeta-Aqueveque C, Robles MdR, Cattan PE. Helmintofauna del roedor *Abrothrix olivaceus* (Sigmodontinae) en áreas sub-urbanas de Santiago de Chile. *Parasitología Latinoamericana.* (2007) 62**:** 134-41. doi: 10.4067/S0717-77122007000200006

38. Landaeta-Aqueveque C, Notarnicola J, Correa JP, Yáñez-Meza A, Henríquez A, Cattan PE, et al. First record of *Litomosoides pardinasi* (Nematoda: Onchocercidae) in native and exotic rodents from Chile. *Revista Mexicana de Biodiversidad.* (2014) 85**:** 1032-7. doi: 10.7550/rmb.44711

39. Landaeta-Aqueveque C, Robles MDR, Cattan PE. The community of gastrointestinal helminths in the housemouse, *Mus musculus*, in Santiago, Chile. *Parasitología Latinoamericana.* (2007) 62**:** 165-9. doi: 10.4067/S0717-77122007000200010

40. Quentin JC, Courtin SS, Fontecilla JA. *Octdonthoxys gigantea* n. gen. N. sp. Nuevo nematodo Oxyurinae parásito de un roedor caviomorfo de Chile. *Boletín Chileno de Parasitología.* (1975) 30**:** 21-5.

41. Quentin JC. Oxyure de Rongeurs: II. Essai de classification des oxyures Heteroxynematidae. *Memoires du Museum National d' Histoire Naturelle, Zoologie, Serie A* (1975) 94**:** 51-96.

42. Torres P. Hallazgo de *Syphacla muris* (Yamagutl, 1935) (Nematoda Oxyurldae) en Chile. *Archivos de Medicina Veterinaria.* (1971) 3**:** 3-7.

43. Yáñez-Meza A, Landaeta-Aqueveque C, Quiroga N, Botto-Mahan C. Helminthic infection in three native rodent species from a semiarid Mediterranean ecosystem. *Revista Brasileira de Parasitologia Veterinária.* (2019) 28**:** 119-25. doi: 10.1590/s1984-29612019014

44. Quentin JC, Babero BB, Cattan PE. Helmintofaune du Chile: V. *Syphacia* (*Syphacia*) *phyllotios* n. sp. Nouvel oxyurie parasite d'un rongeur cricetide au Chile. *Bulletin du Muséum National D’Histoire Naturelle 4 série.* (1979) 2**:** 323-7.

45. Veloso-Frias J, Silva-De La Fuente MC, Rubio AV, Moreno L, González-Acuña D, Andrés Simonetti J, et al. Variation in the prevalence and abundance of mites parasitizing Abrothrix olivacea (Rodentia) in the ive forest and Pinus radiata plantations in central Chile. *Hystrix, the Italian Journal of Mammalogy.* (2019) 30: 107-11. doi: 10.4404/hystrix–00171-2019

46. Yáñez-Meza A, Moreno L, Botto-Mahan C. Ectoparasites of the endemic rodent *Abrocoma bennetti* (Hystricomorpha: Abrocomidae) from semiarid Chile. *Gayana.* (2018) 82**:** 94-7. doi: 10.4067/S0717-65382018000100094

47. Lareschi M, González-Acuña D. Acari, Laelapidae (ectoparasite mites), central and southern Chile. *Check List.* (2010) 6: 546-8. doi: 10.15560/6.4.546

48. Barriga OO. Hallazgo en Chile de *Liponyssus bacoti* (Hirst, 1913) (Acarina, Dermanyssidae). *Boletín Chileno de Parasitología.* (1965) 20**:** 30-3.

49. Silva-de la Fuente C (2019). *El complejo Ornithonyssus bacoti (Acari: Mesostigmata) de roedores de Chile: diversidad genética, variaciones morfológicas y patógenos asociados.* Ph. D. Ph. D. Thesis, Universidad de Concepción.

50. Lee Goff M, Webb JP. A new genus and species of Leeuwenhoekiinae (Acari: Trombiculidae) from rodents collected in Chile, and a key to the new world genera of Leeuwenhoekiinae. *International Journal of Acarology.* (1989) 15**:** 75-8. doi: 10.1080/01647958908683828

51. Webb JP, Bennett SG, Loomis RB. A new genus and species of trombiculid mite (Acari) from a Chilean rodent (Mammalia: Cricetidae). *International Journal of Acarology.* (1986) 12**:** 83-5. doi: 10.1080/01647958608683446

52. Silva-de la Fuente MC, Casanueva ME, Moreno Salas L, González-Acuña D. A new genus and species of chigger mite (Trombidiformes: Trombiculidae) from *Loxodontomys pikumche* (Rodentia: Cricetidae) in Chile. *Zootaxa.* (2015) 4092**:** 426-30. doi: 10.11646/zootaxa.4092.3.8

53. Silva-de la Fuente C, Stekonikov AA, Weitzel T, Beltrami E, Martínez-Valdevenito C, Abarca K, et al. Chigger mites (Acariformes: Trombiculidae) of Chiloe Island, Chile, with descriptions of two new species and new data on the genus *Herpetacarus*. *Journal of Medical Entomology.* (2021). doi: 10.1093/jme/tjaa258

54. Brennan JM, Goff ML. Three new monotypic genera of chiggers (Acari: Trombiculidae) from South America. *Journal of Medical Entomology.* (1978) 14**:** 541-4. doi: 10.1093/jmedent/14.5.541

55. Goff ML, Webb JPJ. A new species of *Paraguacarus* (Acari: Trombiculidae) from a degu (Mammalia: Rodentia) collected in Chile. *Bulletin of the Society for Vector Ecology.* (1989) 14**:** 93-4.

56. Sikora B, Bochkov A. Fur mites of the family Listrophoridae (Acariformes: Sarcoptoidea) associated with South American sigmodontine rodents (Cricetidae: Sigmodontinae). *Acta parasitologica.* (2012) 57**:** 388-96. doi: 10.2478/s11686-012-0046-1

57. Guglielmone AA, Nava S, Bazán-León EA, Vásquez RA, Mangold AJ. Redescription of the male and description of the female of *Ixodes abrocomae* Lahille, 1916 (Acari: Ixodidae). *Systematic Parasitology.* (2010) 77**:** 153-60. doi: 10.1007/s11230-010-9262-y

58. Muñoz-Leal S, Marcili A, Fuentes-Castillo D, Ayala M, Labruna MB. A relapsing fever *Borrelia* and spotted fever *Rickettsia* in ticks from an Andean valley, central Chile. *Experimental and Applied Acarology.* (2019) 78**:** 403-20. doi: 10.1007/s10493-019-00389-x

59. Muñoz-Leal S, Venzal JM, Nava S, Marcili A, González-Acuña D, Martins TF, et al. Description of a new soft tick species (Acari: Argasidae: *Ornithodoros*) parasite of *Octodon degus* (Rodentia: Octodontidae) in northern Chile. *Ticks and Tick-borne Diseases.* (2020) 11**:** 101385. doi: 10.1016/j.ttbdis.2020.101385

60. Emerson KC, Price RD. Abrocomophagidae (Mallophaga: Amblycera), a new family from Chile. *The Florida Entomologist.* (1976) 59**:** 425-8. doi: 10.2307/3494196

61. Price R, Timm R. Review of the chewing louse genus *Abrocomophaga* (Phthiraptera: Amblycera), with description of two new species. *Proceedings of the Biological Society of Washington.* (2000) 113**:** 210-7.

62. Durden LA, Gomez MS. *Abrocomaphthirus chilensis* (Gomez), new combination (Phthiraptera-Anoplura), an ectoparasite of the Chilean rodent *Abrocoma bennetti* (Abrocomidae). *Parasite.* (2000) 7**:** 331-2. doi: 10.1051/parasite/2000074331

63. Gomez MS. Two Anoplura species from rodents in Chile: *Hoploplerura andina* Castro, 1981 (Hoplopleuridae) from *Geoxus valdivianus* (Cricetidae) and *Eulinognathus chilensis* n. sp. (Polyplacidae) from *Abrocoma bennetti* (Abrocomidae). *Research and Reviews in Parasitology.* (1998) 58**:** 49-54.

64. Moreno-Salas L, Castro DdC, Torres-Murua JC, González-Acuña D. Phthiraptera (Amblycera and Anoplura) parasites of the Family Octodontidae, Ctenomidae and Abrocomidae (Mammalia: Rodentia) from Chile. *Rudolstädter Naturhistorische Schriften.* (2005) 13**:** 115-8.

65. Castro DdC, Cicchino A. Las especies del género *Gyropus* Nitzsch, 1818 (Phthiraptera: Gyropidae) parásitas de Octodontidae (Mammalia: Rodentia). *Revista Chilena de Historia Natural.* (2002) 75**:** 293-8. doi: 10.4067/S0716-078X2002000200003

66. Ewing HE. On the taxonomy, biology, and distributuion of the biting lice of the family Gyropodidae. *Proceedings of the U. S. National Museum.* (1924) 63**:** art. 20.

67. González-Acuña D, Del C. Castro D, Moreno-Salas L. Contribucion al conocimiento de los Phthiraptera (Anoplura, Hoplopleura) parasitos de roedores en Chile. *Gayana.* (2003) 67**:** 117-9. doi: 10.4067/s0717-65382003000100014

68. González-Acuña D, Castro D, Mey E, Torres-Mura J. New records of Sucking lice (Insecta: Phthiraptera: Anoplura) on rodents (Mammalia: Rodentia: Muridae) from Chile. *Mastozoología Neotropical.* (2005) 12.

69. Castro DC. Contribución al conocimiento de los Anoplura neotropicales. *Revista de la Sociedad de Entomología de Argentina.* (1981) 40**:** 231-6.

70. Macchiavello A. Siphonaptera de la costa Sur-Occidental de América (Primera lista y distribucion zoo-geográfica). *Boletín de la Oficina Sanitaria Panamericana.* (1948) 27**:** 412-60.
